# Active Precipitation of Radiation Belt Electrons Using Rocket Exhaust Driven Amplification (REDA) of Man‐Made Whistlers

**DOI:** 10.1029/2022JA030358

**Published:** 2022-06-01

**Authors:** P. A. Bernhardt, M. Hua, J. Bortnik, Q. Ma, P. T. Verronen, M. P. McCarthy, D. L. Hampton, M. Golkowski, M. B. Cohen, D. K. Richardson, A. D. Howarth, H. G. James, N. P. Meredith

**Affiliations:** ^1^ Geophysical Institute University of Alaska Fairbanks AK USA; ^2^ Department of Atmospheric and Oceanography Science UCLA Los Angeles CA USA; ^3^ Center for Space Physics Boston University Boston MA USA; ^4^ Sodankylä Geophysical Observatory University of Oulu Sodankylä Finland; ^5^ Space and Earth Observation Centre Finnish Meteorological Institute Helsinki Finland; ^6^ Department of Earth and Space Sciences University of Washington Seattle WA USA; ^7^ Department of Electrical Engineering University of Colorado Denver Denver CO USA; ^8^ School of Electrical and Computer Engineering Georgia Institute of Technology Atlanta GA USA; ^9^ Department of Physics and Astronomy University of Calgary Calgary AB Canada; ^10^ British Antarctic Survey Natural Environment Research Council Cambridge UK

**Keywords:** active space experiments, parametric amplifier, wave particle interactions, amplified whistler wave

## Abstract

Ground‐based very low frequency (VLF) transmitters located around the world generate signals that leak through the bottom side of the ionosphere in the form of whistler mode waves. Wave and particle measurements on satellites have observed that these man‐made VLF waves can be strong enough to scatter trapped energetic electrons into low pitch angle orbits, causing loss by absorption in the lower atmosphere. This precipitation loss process is greatly enhanced by intentional amplification of the whistler waves using a newly discovered process called rocket exhaust driven amplification (REDA). Satellite measurements of REDA have shown between 30 and 50 dB intensification of VLF waves in space using a 60 s burn of the 150 g/s thruster on the Cygnus satellite that services the International Space Station. This controlled amplification process is adequate to deplete the energetic particle population on the affected field lines in a few minutes rather than the multi‐day period it would take naturally. Numerical simulations of the pitch angle diffusion for radiation belt particles use the UCLA quasi‐linear Fokker Planck model to assess the impact of REDA on radiation belt remediation of newly injected energetic electrons. The simulated precipitation fluxes of energetic electrons are applied to models of D‐region electron density and bremsstrahlung X‐rays for predictions of the modified environment that can be observed with satellite and ground‐based sensors.

## Key Points

- Rocket exhaust driven amplification (REDA) produces the strongest whistler amplitudes measured in space from ground‐based very low frequency (VLF) transmitters
- REDA intensified whistlers can rapidly scatter radiation belt electrons into the loss cone in minutes rather than days
- Effects of REDA induced electron losses to the atmosphere include enhanced X‐rays, airglow, VLF propagation changes, and radio absorption

## 1 Introduction

The natural and artificial production of high‐intensity whistler waves in space is of interest because of their interaction with radiation belt particles. Lightning bursts excite large amplitude pulses of electromagnetic radiation that couple through the bottom of the ionosphere, are ducted along magnetic field lines, and interact with the Earth's radiation belts to produce lightning‐induced electron precipitation (LEP). In this interaction process, amplified whistler, triggered emissions, and enhancements in the electron density of the lower ionosphere have been observed (Voss et al., 1984). LEP is also produced by nonducted (magnetospherically reflected) whistlers (Bortnik et al., 2006a, 2006b; Lauben et al., 2001). Other natural whistler mode emissions include chorus, plasmaspheric hiss, and magnetosonic waves. Chorus waves are strong, natural very low frequency (VLF) emissions generated in the inner magnetosphere during storms and substorms and can dramatically affect electron acceleration and loss timescales (Ozaki et al., 2019). They play a major role in radiation belt dynamics contributing to both the acceleration and loss of relativistic electrons (Bortnik & Thorne, 2007). Plasmaspheric hiss is another important, natural, magnetospheric emission which is observed in the plasmasphere and plasmaspheric plumes. It is largely responsible for the formation of the slot region (for example, Lyons & Thorne, 1973; Lyons et al., 1972) and the quiet time decay of outer radiation belt electrons (Meredith et al., 2006). Magnetosonic waves, which are observed both inside and outside the plasmapause, can also contribute to both the acceleration and loss of radiation belt particles (Horne et al., 2007; Meredith et al., 2009).

In addition, ambient power‐line harmonics (Fedorov et al., 2021; Park & Helliwell, 1978), intentional VLF transmissions from terrestrial power lines and high‐power VLF transmitters can leak into space where they may interact with energetic electrons in the Earth's magnetosphere (Hua et al., 2020; Ma et al., 2017; Ross et al., 2019). Several man‐made facilities have been developed to study this wave‐particle‐interaction process including VLF transmitters (Helliwell, 1977, 1988), high‐power HF facilities for modulations of natural ionosphere currents in the ionosphere (Guo et al., 2021), large satellite antennas driven by high power signal generators, electron beams that are modulated at VLF rates, and high‐speed neutral injections that rapidly photoionize in sunlight (Borovsky & Delzanno, 2019; Sotnikov et al., 2018). These techniques require dedicated, expensive engineering efforts for design, construction, and testing before they are deployed on the ground or in space. The VLF waves currently injected into space are reduced to ineffective levels by nonlinear losses (Galinsky et al., 2011; Mishin et al., 2010) and propagation spreading.

A new, currently available process for amplification of whistler signals to useful levels involves transferring energy from pickup ions in a rocket exhaust plume to the electromagnetic (EM) waves. The process uses lower‐hybrid waves from an artificial source to power the amplifier. A rocket engine in space can excite large‐amplitude lower hybrid waves by charge exchange of the plume molecules with ambient oxygen ions in the ionosphere (Bernhardt et al., 2012; Bernhardt & Sulzer, 2004). The rocket engine burn yields energetic molecules by the reaction $N_{2}O_{4}+2N_{2}H_{4}\to 4H_{2}O^{*}+3N_{2}^{*}$, where the asterisk indicates molecules moving at between 4 and 10 km/s, depending on orientation of the rocket nozzle relative to the orbit velocity vector. Streaming exhaust molecules encounter atomic oxygen ions and hypersonic ions are created by $H_{2}O^{*}+O^{+}\to H_{2}O^{+*}+O,N_{2}^{*}+O^{+}\to {\text{NO}}^{+*}+N$. The resulting pickup ions form into a ring‐beam distribution that excites a lower hybrid wave instability as described in the next section.

Sections 3 and 4 describe the utility of the whistler amplification process with simulations of induced electron precipitation, enhanced optical and X‐ray emissions, radio absorption in the D‐region, and changes in the VLF propagation constrained in the Earth‐ionosphere waveguide (EIWG). These changes will provide indirect measurements of the amplification using both ground and satellite instruments. The conclusions and recommendations for future research are given in the last section.

## 2 Whistler Wave Amplification in Space

The rocket exhaust driven amplification (REDA) technique described here uses existing technologies to amplify signals from existing ground‐based transmitters with dedicated firings of rocket motors in low‐earth‐orbit. The technique converts the ambient atomic oxygen ions in the topside ionosphere to an activated plasma region with pickup ions gyrating around the magnetic field lines. Whistler waves passing though this region are parametrically amplified by converting the energy of the gyrating ions into intense electromagnetic signals.

**Figure 1:**
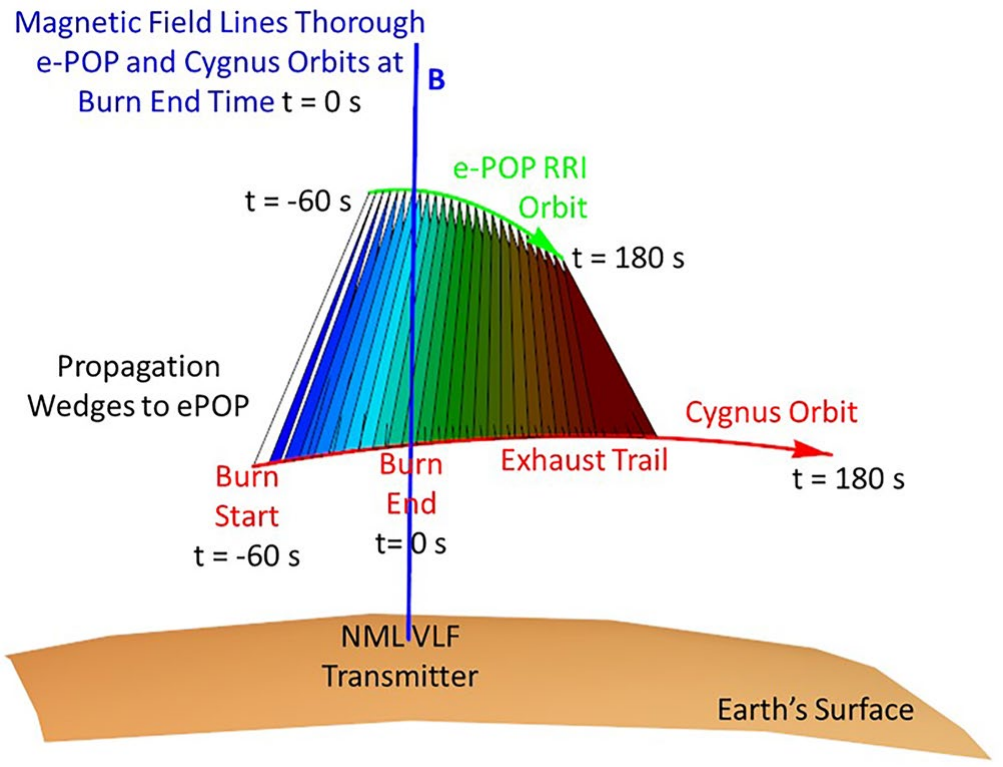
Experimental geometry for amplification of ground‐based very low frequency (VLF) signals by rocket exhaust injections in space. The NML transmitter in North Dakota continuously broadcasts 25.2 kHz signals that enter the ionosphere as low‐amplitude whistler waves. When the whistlers pass though the rocket engine plume, they become amplified for reception on by the plasma wave sensor on the e‐POP payload simultaneously passing over the interaction region.

The first demonstration of REDA of whistler mode waves occurred on 26 May 2020 by transferring energy from pickup ions in a rocket exhaust plume to EM waves (Bernhardt et al., 2021). The source of coherent VLF waves was the Navy NML Transmitter at 25.2 kHz located in LaMoure, North Dakota. The topside ionosphere at 480 km altitude became an amplifying medium with a 60 s firing of the Cygnus BT‐4 engine during the NG‐13 Mission after undocking from the International Space Station (ISS). The rocket engine injected exhaust near 500 km altitude as a neutral cloud moving perpendicular to field lines. The NML transmitter, the Cygnus burn, and the VLF Radio Receiver Instrument (RRI) on e‐POP/SWARM‐E (James et al., 2015) at 1,000 km altitude were all on the same field lines within 10 s of the burn. Charge exchange between the ambient O+ ions and the hypersonic water molecules in the exhaust produce H_2_O^+^ ions in a ring‐beam velocity distribution (Bernhardt et al., 2021). The charge exchange rate is velocity dependent with a value of 3.2 × 10^−9^ cm^3^/s (Bernhardt, 1987; X. Li et al., 1995). A diagram of the experiment is shown in Figure 1.

**Figure 2:**
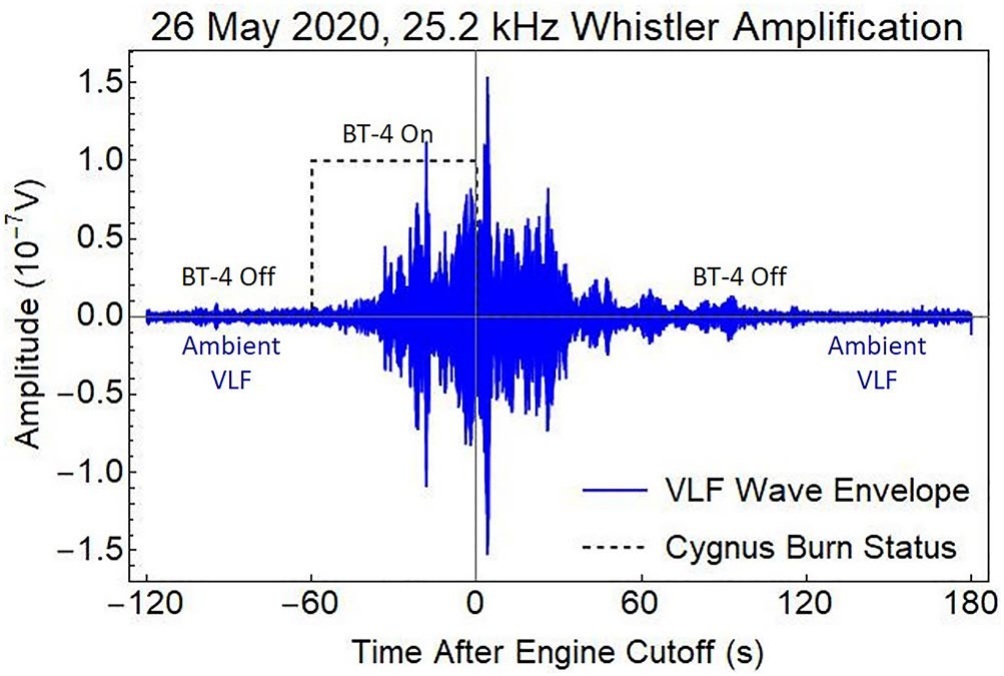
Demodulated signal showing between 20 and 30 dB amplification of the 25.2 kHz ground transmissions from the NML very low frequency (VLF) station directly below Cygnus orbiting at 500 km altitude. The measured potential on a 6 m boom of the Radio Receiver Instrument (RRI) antenna is converted into low‐frequency electric fields assuming a boom sample distance of 3 m. The peak signal strength of 0.15 μV on the dipole‐antenna channel A is influenced by its orientation.

The RRI recorded the enhanced VLF signals on channels A and B of crossed dipole antennas at 1,000 km altitude. The SWARM‐E/e‐POP satellite passed through the whistler propagation cone around the magnetic field relative to the Cygnus exhaust cloud. The 25.2 kHz VLF signal from NML was amplified by 30 dB as observed by channel A of the RRI (Figure 2). The flow rate from the BT‐4 engine was 150 g/s so the 60 s burn released 9 kg of exhaust over a 440 km horizontal orbit trajectory with a satellite velocity of 7.3 km/s. The injection speed relative to the background atmosphere was 4.3 km/s for a 3.0 km/s plume in the satellite wake direction.

**Figure 3:**
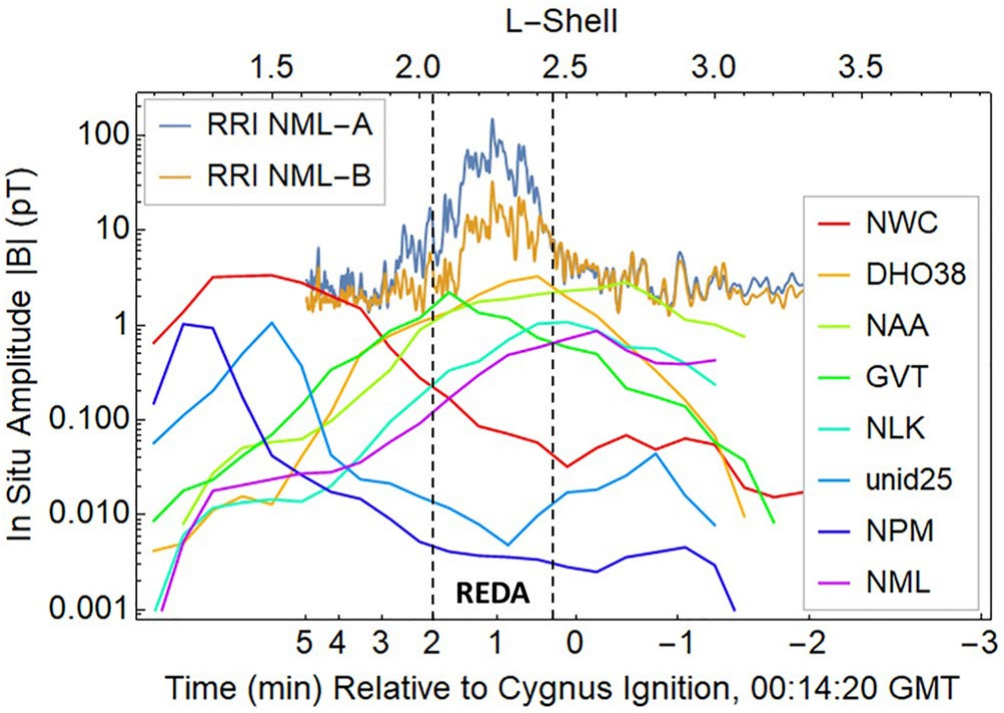
Measured very low frequency (VLF) amplitudes of whistler waves from ground transmitters using (a) 5 yr averages from the Van Allen Probe A and (b) instantaneous observations of the A and B channels of Radio Receiver Instrument (RRI) on SWARM‐E/e‐POP. Differences between the two NML data sets are attributed to magnetic local time (MLT) dependence on D‐region absorption and variations in satellite positions relative to the VLF transmitters. The unamplified signal from the NML transmitter in North Dakota is the lowest of the averaged data at mid‐latitudes. The amplified NML signal during the REDA event has the largest amplitude of coherent VLF signals ever observed in the ionosphere.

The significance of the rocket‐burn amplification is found by comparing the NG‐13 experiment with previous observations of ground‐based VLF transmissions in space. The Electric and Magnetic Field Instrument Suite and Integrated Science (EMFISIS) sensor on the Van Allen Probe A satellite was used to collect plasma wave data (Kletzing et al., 2013) including signals from ground‐based VLF transmitters. These data were analyzed to provide the 5 yr average intensity over magnetic local time (MLT) and sensor altitudes at L‐shells in the 1–3.5 range (Meredith et al., 2019). The amplified VLF signals for the NML station provided by the two channels of the RRI at 1,050 km altitude are compared with the unamplified VLF signals for the eight strongest VLF transmitters observed by the EMFISIS in Figure 3 (bottom eight curves).

The NML signal strength outside of the REDA region from RRI is about eight‐times larger than the 5 yr average value from EMFISIS. This is because the NG‐13 REDA experiment occurred near 18:00 MLT directly over the transmitter at 1,050 km altitude whereas the averaged EMFISIS observations include all local times when the satellite is far (in longitude and altitude) from the transmitter.

Within the REDA region, Cygnus rocket burn enhances the NML whistler amplitudes (top two curves with channels A and B of RRI data in Figure 3) well above average ambient VLF levels. The peak REDA amplitude is 270 pT, including corrections for the saturation of the RRI receiver (Bernhardt et al., 2021). The rocket‐burn amplification of the most powerful transmitters (NWC, DH038, NAA), which are three times stronger than NML, could produce whistler waves amplitudes over 1,000 pT. This article investigates the impact of these strong waves on the trapped energetic electrons in the radiation belts.

A preexisting coherent ELF signal at 300 Hz was amplified by 50 dB during and after the Cygnus burn. Extremely strong coherent emissions and quasi‐periodic bursts in the 300–310 Hz frequency range lasted for 200 s after the release. The excitation of an ELF whistler cavity may have lasted even longer, but the orbit of the SWARM‐E/e‐POP moved the RRI sensor away from the wave emission region. The rocket‐burn amplified 300 Hz ELF waves may have gained even more energy by cyclotron resonance with radiation belt electrons while ducted between geomagnetic‐conjugate hemispheres (Bernhardt et al., 2021).

Another report on intense whistlers was made during the STEREO mission (Cattell et al., 2008). The whistler waves observed with STEREO show 240 mV/m electric field transients in the radiation belts. The REDA observations by e‐POP/RRI is 2–20 mV/m coherent whistlers at 300 Hz and 24.2 kHz for a duration of 60 s in the ionosphere. Both these type waves in the radiation belts can produce enhanced scatter into the loss cone and particle acceleration to higher energies (Hua et al., 2022). Future studies should determine the relative effects of a short burst of transient whistlers with high wave‐normal angles or a long continuous source of coherent whistlers that can be trapped in ducts for field‐aligned wave‐normal propagation.

One approach for comparisons of intense coherent waves (REDA) to transient naturally occurring waves using a quasi‐linear model is to compute the energy in the wave during the interactions. A 0.1 s burst at 240 mV/m has 25% of the energy of a 60 s VLF tone with 20 mV/m amplitude. The REDA approach to radiation belt interactions studies can produce effects comparable and even exceeding natural whistler events.

**Figure 4:**
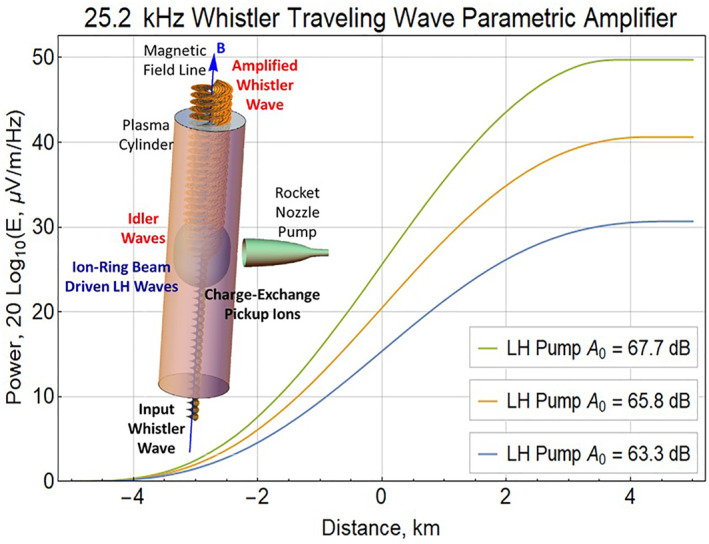
Design and performance of the rocket exhaust driven amplification (REDA) physical device. (inset) The amplitude of the right‐hand circular polarization is represented as a spiral with growth after passing though the activated region with the ion‐ring beam distribution. The curves show the lower hybrid pump control of whistler amplification and saturation levels. The units for pump amplitude A0 are the same as the dB power scale for the whistler wave.

Experimental measurements during the Cygnus NG‐13 Mission demonstrate that a wide range of ELF and VLF frequencies can be amplified using a rocket exhaust injection of water vapor. The device, shown in Figure 4, used to amplify the ELF/VLF signals is called a whistler traveling wave parametric amplifier (WTWPA; Bernhardt, 2021). The amplification process uses an input whistler mode signal, a lower‐hybrid pump wave, and a daughter idler wave, to provide an amplified whistler wave. The rocket nozzle is directed perpendicular to the magnetic field lines to yield a ring‐beam distribution of ions that gyrate around the magnetic field (B). The perpendicular ion motion excites a broad spectrum of pump, lower hybrid (LH) waves by a lower‐hybrid instability (Akimoto et al., 1986; Winske & Daughton, 2012). The parametric conversion process relies on frequency and wavenumber matching conditions (*ω*_*0*_ = *ω*_*1*_ + *ω*, ***k***_*0*_ = ***k***_*1*_ + ***k***) for the electrostatic pump wave (*ω*_*0*_, ***k***_*0*_) to decay into a whistler wave (*ω*_*1*_, ***k***_*1*_) and another lower hybrid wave (*ω*, ***k***).

Computation results of REDA gain with three pump amplitudes are illustrated by Figure 4, using the whistler frequency and plasma parameters given for the Cygnus NG‐13 experiment (Bernhardt, 2021; Bernhardt et al., 2021). The input amplitude of the wave is set at 0 dB and, depending on the selected pump amplitude; the waves grow spatially after passing through 10 km of activated plasma to obtain gains near 30, 40, and 50 dB. The shape of the LH wave distribution is a cosine‐squared envelope of the pump wave over 10 km range. The wave amplitudes grow monotonically at rates determined by the three values of lower‐hybrid pump amplitude (A_0_) as the whistlers propagate through the region with an ion‐ring plasma distribution. The whistler amplitude for the highest gain REDA is flattened as it exits the active region because the pump is depleted by full transference of energy to the amplified wave. Thus, the WTWPA has no threshold for amplification but it has a saturation limit. The magnitude of the whistler mode when the amplifier is saturated should be strongest near the center of the exhaust cloud where exhaust pickup ion fraction is the greatest and the pump amplitude is expected to be at a maximum. Based on experimental measurements during the NG‐13 Cygnus mission and these WTWPA computations, the amplification factor of the whistler signals from ground‐based transmitters will be taken in the range of 30 through 50 dB, representing typical range of REDA conditions. The specific gains at 30, 40, and 50 dB are taken to represent respective whistler wave amplitudes of 150, 500, and 1,500 pT.

## 3 Impact of REDA on Energetic Electrons

The flux density of energetic electrons in the radiation belts can be rapidly depleted with localized amplification of ambient whistler signals. The differential number flux of precipitating radiation belt electrons is enhanced with the intensified whistler wave signals in space when rocket engines burn directly above ground‐based VLF transmitters. The amplified waves pitch angle scatter trapped radiation into the magnetic field loss cone. This REDA process intentionally drains energetic electrons from the radiation belts. The effectiveness of REDA in space will be simulated using a quasi‐linear, Fokker‐Planck model (Bortnik et al., 2006a, 2006b; Hua et al., 2020).

**Figure 5:**
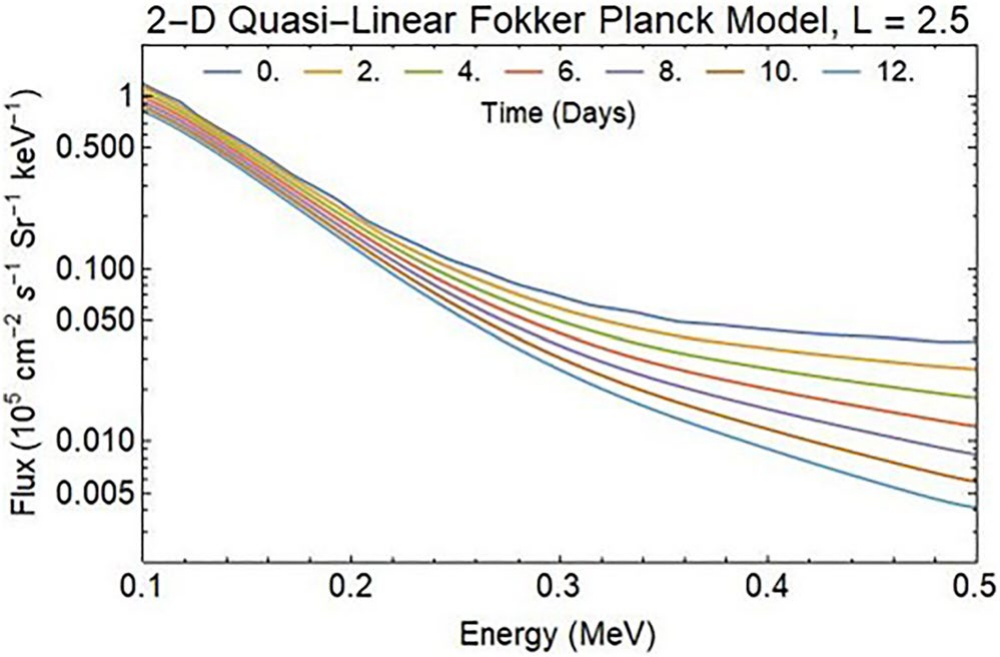
Impact of lightning, hiss, and unamplified very low frequency (VLF) on the differential electron fluxes in the post substorm radiation belts as simulated by Hua et al. (2020).

Enhancements in the drift‐loss cone electron fluxes have been associated with nighttime VLF wave transmissions from the ground (Gamble et al., 2008; Kulkarni et al., 2008; Sauvaud et al., 2019) by cyclotron resonance enhancement of pitch angle diffusion in the radiation belts. These transmitter‐induced precipitation features, called “wisps” in the observations, are only observed at night (i.e., when the D‐region absorption of VLF is low) and for 1.4 < L 1.8 magnetic field lines (i.e., where field‐aligned ducts occur; Sauvaud et al., 2019). These same conditions are used in the quasi‐linear, Fokker‐Planck model studies for the formation of the localized‐depletion of electrons by REDA in the inner radiation belt. Spatially localized amplification of whistler waves are multiple VLF transmitters (such as NML at 25.2 kHz) represented statistically with a 2 kHz bandwidth (Ma et al., 2017) in the region 2.4 < *L* < 2.6. The Fokker‐Planck simulations (10–600 keV) use identical initial and background conditions representing February–March 2016 provided by data from the Radiation Belt Storm Probes Ion Composition Experiment (RBSPICE) onboard the Van Allen Probes (Hua et al., 2020). Statistical wave models for plasmaspheric hiss, magnetosonic waves, and lightning generated whistlers (LGW) are from W. Li et al. (2015), Ma et al. (2016), and Green et al. (2020), respectively. The model uses a dipolar magnetic field model and empirical electron density model given by Ozhogin et al. (2012). The ambient environment of LGW, hiss, and unamplified VLF transmitters produces a slow but continuous draining of the trapped radiation belts (Figure 5). The upper boundary at 500 keV uses a fixed flux of 0.035 × 10^5^ cm^−2^ s^−1^ Sr^−1^ keV^−1^.

Pitch angle and momentum transfer in the rocket exhaust driven amplification (REDA) zone yields scattering into the precipitation loss cone. Once inside the loss cone, the timescale for loss, τ, is one‐quarter the particle bounce period.

The REDA gain factors of 0, 30, and 50 dB will be used to estimate impacts on the energetic electron populations in the radiation belts. The trapped electron density distribution is computed using the Fokker‐Planck diffusion equation:

(1) $$\begin{array}{l} \frac{\partial f}{\partial t}=\frac{1}{G}\frac{\partial }{\partial \alpha }\left(D_{\alpha \alpha }G\frac{\partial f}{\partial \alpha }\right)+\frac{1}{G}\frac{\partial }{\partial \alpha }\left(pD_{\alpha p}G\frac{\partial f}{\partial p}\right)\\ +\frac{1}{G}\frac{\partial }{\partial p}\left(pD_{p\alpha }G\frac{\partial f}{\partial \alpha }\right)+\frac{1}{G}\frac{\partial }{\partial p}\left(p^{2}D_{pp}G\frac{\partial f}{\partial p}\right)-\frac{f}{\tau } \end{array}$$

where *f*(*α*, *p*) is the phase‐space density, *α* is the equatorial pitch angle, *p* = *γm*_*e*_v is the momentum, and *G* = *p* ^*2*^ *S*(*α*) sin*α* cos*α* is a scale factor related to the bounce period (Hua et al., 2020). The bounce averaged coefficients (*D*_*αα*_, *D*_*αp*_ *= D*_*pα*_, *D*_*pp*_) are enhanced by the amplified whistler waves. The diffusion coefficients are calculated using statistical frequency spectrum of VLF transmitter waves (Ma et al., 2017), considering up to 10 orders of resonant harmonics with electrons.

**Figure 6:**
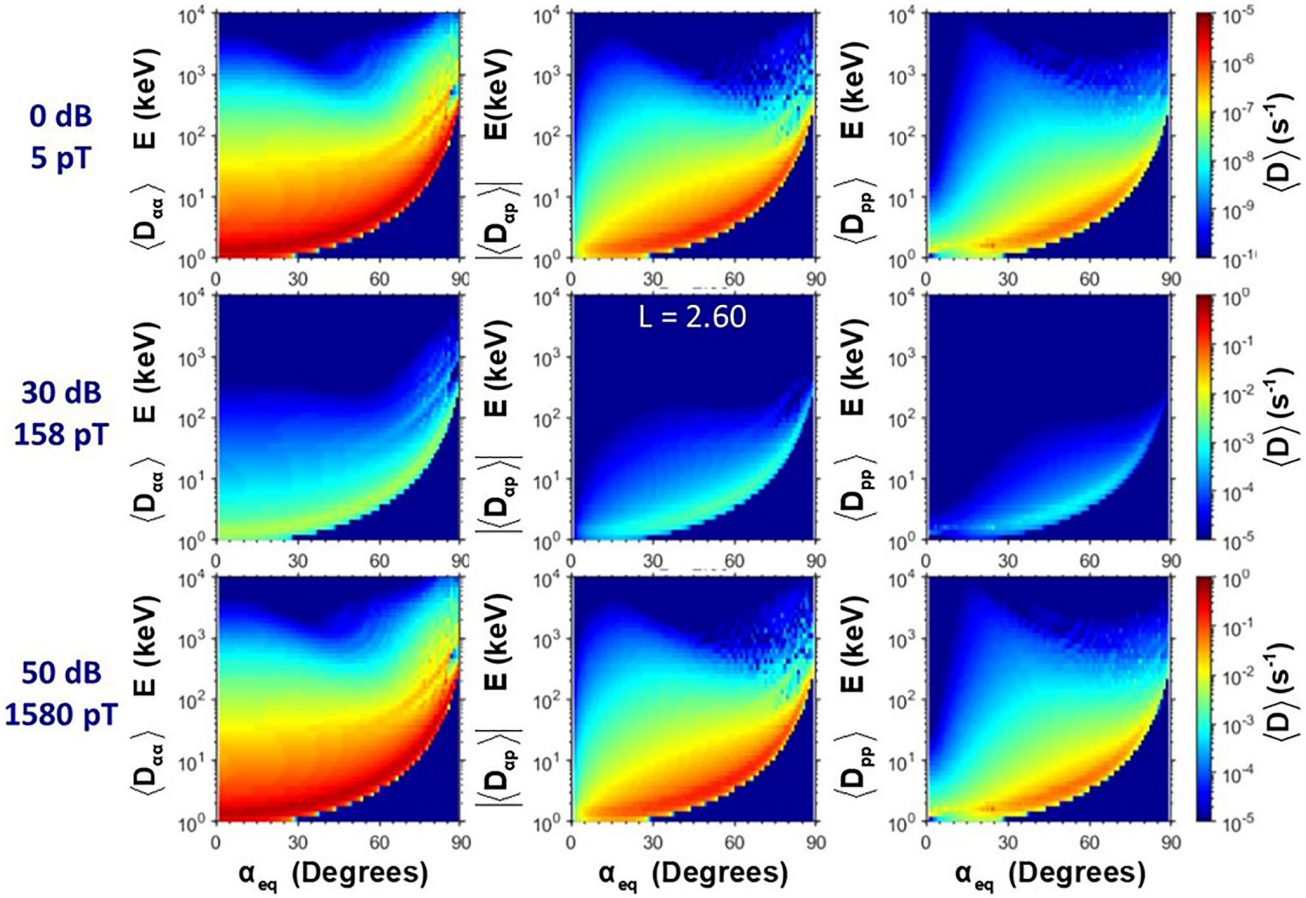
Quasi‐linear electron diffusion coefficients due to very low frequency (VLF) transmitter waves (25.2 kHz) at *L* = 2.6 with different rocket exhaust driven amplification in dB and corresponding whistler amplitude, Bw, in pT. The top simulations for the diffusion in the ambient, unamplified environment have a maximum pitch angle diffusion rate of 10^−5^ s^−1^. The coefficients using 30 dB for the 25.2 kHz amplification are shown in the middle row with the maximum diffusion rate to 10^−2^ s^−1^. The bottom row with a 50 dB rocket exhaust driven amplification (REDA) enhancement in the VLF amplitude yields a maximum diffusion rate approaching 1 s^−1^. Note that the color scale for the diffusion coefficients is different for each row.

The enhanced diffusion at *L* = 2.6 is seen in Figure 6; similar changes in distributions in diffusion are found at all L‐shells affected by the amplified whistlers. Increased diffusion yields a rapid change in the pitch angle, momentum, and energy distributions of the particles. The primary time‐dependent loss of energetic electrons occurs for those with pitch angles *α* < *α*_*C*_ where the equatorial pitch angle of the bounce loss cone, *α*_*C*_, is given in a dipole magnetic field by sin^2^ *α*_*C*_ = (4*L* ^6^ − 3*L* ^5^)^−1/2^. The equatorial electron flux is related to the phase‐space density by *j*(*α*, *p*) = *f*(*α*, *p*)*p* ^*2*^ and can be measured by particle detectors on satellites.

**Figure 7:**
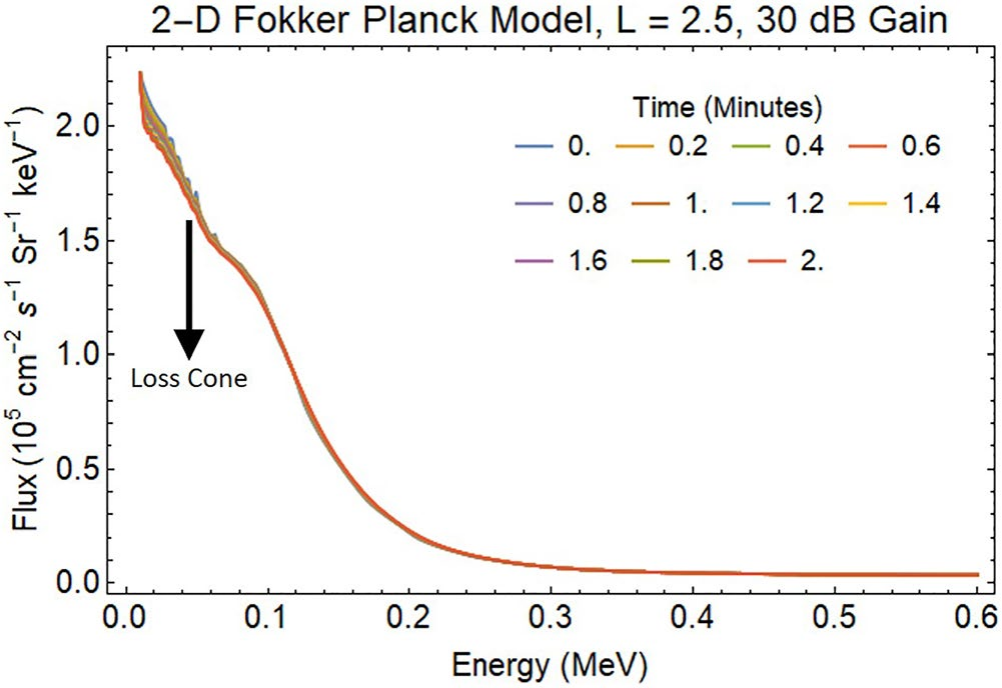
Impact of 2 min BT‐4 wake burn on radiation belt electrons simulated by the UCLA 2‐D Fokker‐Planck Model for 30 dB rocket exhaust driven amplification of whistler waves.

Starting with a quasi‐equilibrium distribution of background particles that slowly decays in the radiation belts between *L* = 1.5–3, amplification factors of 0, 30, and 50 dB are employed by the Fokker‐Planck model for a two‐minute period in three separate runs. The results for the 30 dB REDA are illustrated in Figure 7. The reductions in energetic electron fluxes are primarily below 100 keV energies. Figures 5 and 7 show that 2 min of 30 dB REDA produces a 0.2 × 10^5^ cm^−2^ s^−1^ Sr^−1^ kev^−1^ change trapped flux at *L* = 2.5 that would have taken 2.5‐day in the unamplified plasma environment.

**Figure 8:**
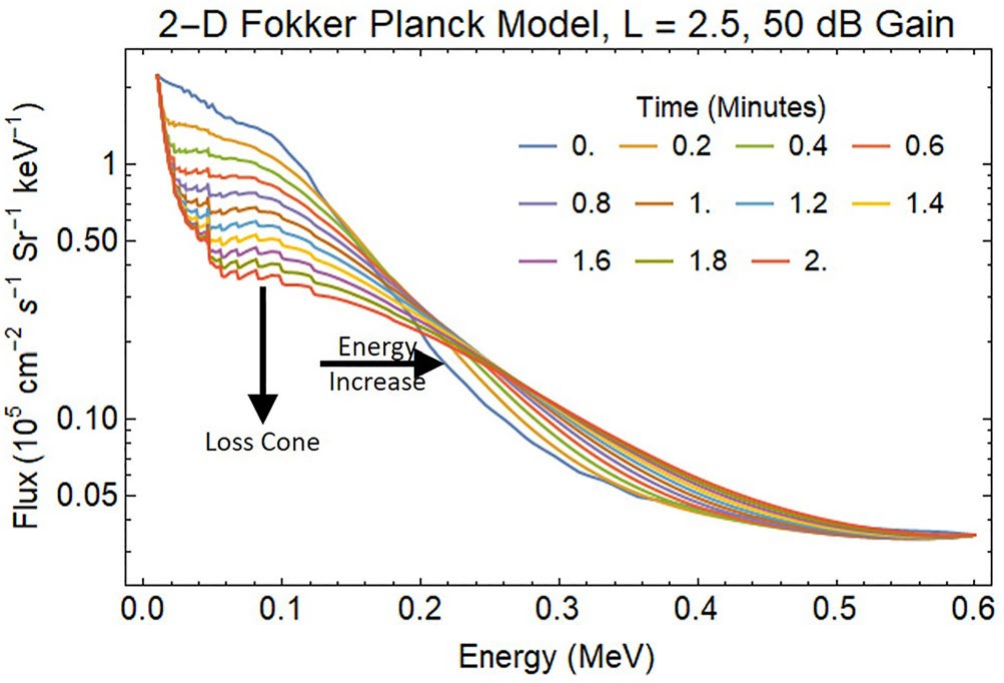
Impact of 2 min BT‐4 wake burn on radiation belt electrons for a 50 dB enhancement in whistler wave power.

Amplification of ambient VLF signals at 50 dB yields a dramatic loss of differential electron fluxes in the radiation belts (Figure 8.). After 2 min of REDA, the whistler waves scatter 80% of the energetic electrons with energies less than 100 keV into the loss cone in the vicinity of the affected field lines. In addition, electrons are scattered to higher energy so that the electron flux above 200 keV is significantly increased. This acceleration process will be the focus of a future article. Enhanced precipitation at higher energies will occur for ELF and VLF frequencies that meet the cyclotron resonance criteria. The REDA can also occur for electromagnetic ion cyclotron (EMIC) waves leading to enhanced precipitation of both ultra‐relativistic electrons and energetic protons.

Scatter of energetic electrons into the loss cone produces an enhancement in precipitation into the lower atmosphere. At energies below 200 keV, simulations show large field‐aligned transport fluxes to the neutral atmosphere. This precipitation flux is found by integration of *j* (*α*, *p*) over equatorial pitch angles less than *α*_*C*_ and by considering flux density increase as the area of the magnetic flux tube is reduced for particles moving down field lines from the equator to the Earth's surface.

**Figure 9:**
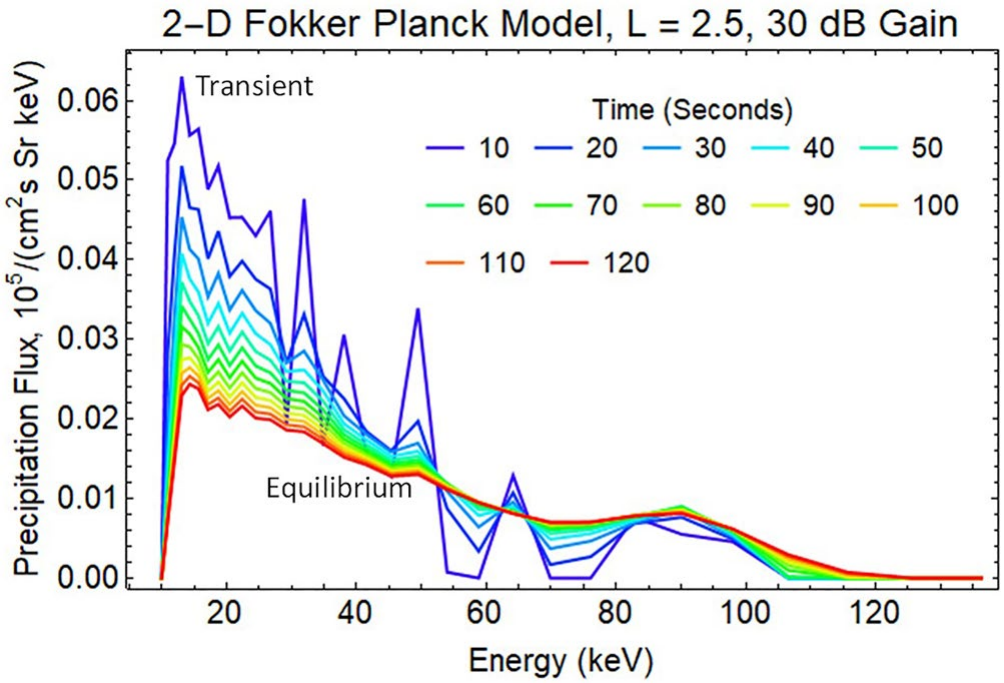
Spectrum of energetic electrons precipitated into the atmosphere by the loss of radiation belt electrons from the 30 dB rocket exhaust driven amplification (REDA) shown in Figure 7. The maximum precipitation flux is about 6 × 10^3^ cm^−3^ s^−1^ Sr^−1^ keV^−1^.

The enhanced precipitating flux for 30 dB amplification of transmitted VLF signals after a REDA burn is displayed in Figure 9. The 120 s of 30 dB REDA initiates a transient in precipitation that relaxes to a near steady state after 100 s. After the simulated REDA is switched off, the enhanced precipitation returns to zero. The actual precipitation flux will respond to a more gradual rise and fall in the amplifications with an envelope similar to the one shown in Figure 2.

**Figure 10:**
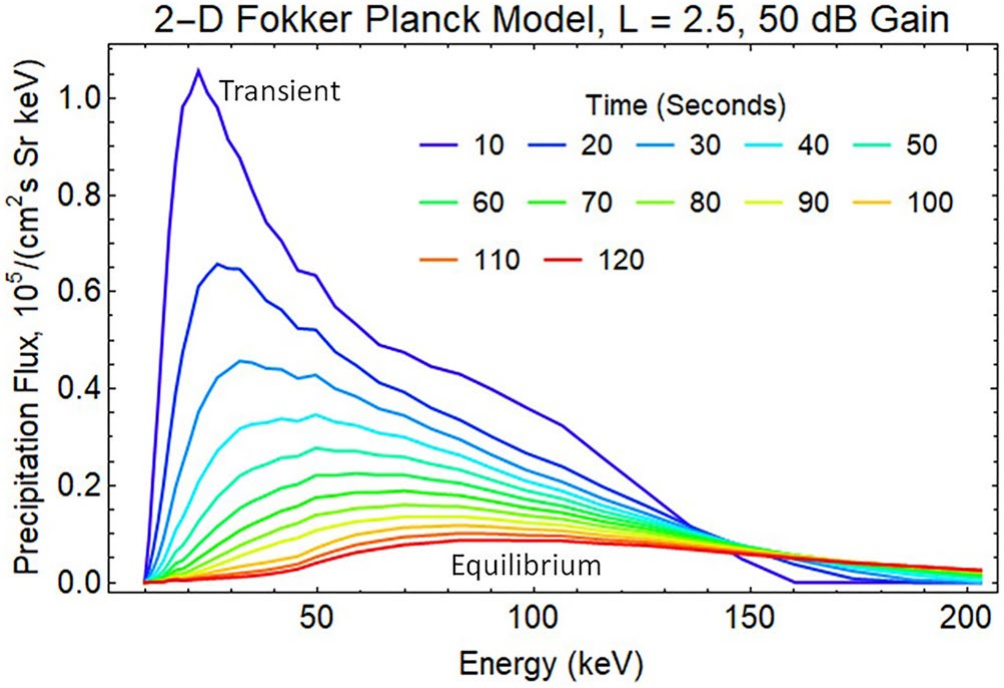
Spectrum of energetic electrons precipitated into the atmosphere by the loss of radiation belt electrons from the 50 dB rocket exhaust driven amplification (REDA) shown in Figure 8. The maximum precipitation flux is about 1 × 10^5^ cm^−3^ s^−1^ Sr^−1^ keV^−1^, which is 17 times the flux in Figure 9.

As expected, the 50 dB REDA with a 1,500 pT whistler wave produces a much larger flux of energetic electrons that precipitate into the lower atmosphere (Figure 10). The large whistler amplitudes from REDA require considering phase trapping during the wave‐particle interaction calculations. Future simulations should use a Vlasov‐Liouville (VL) model, which computes the phase‐space particle distribution function directly using a characteristic‐based solution of the Vlasov equation. Previous work has shown that phase trapping contributes significantly to precipitation when the large‐amplitude wave (>100 pT) is present (Harid et al., 2014).

## 4 Measurement Predictions of REDA Effects

After the burning of a rocket engine over a ground‐based ELF/VLF transmitter, particle precipitation from REDA may be measured in space or on the ground. The full range of radiation belt loss and detection processes is found in the text by Jaynes and Usanova (2020). Direct detection can use satellite energetic particle detectors or plasma wave receivers. This type of measurement is difficult because the satellite sensor must traverse the REDA field line. The only in situ space‐based detection of REDA effects used careful coordination of a Cygnus engine burn and SWARM‐E/RRI instrument operation during the NG‐13 Mission (Bernhardt et al., 2021) as shown in Figure 2.

Indirect detection of REDA can use VLF and HF propagation through enhanced D–region densities, balloon observations with bremsstrahlung X‐ray flux counters, and optical emissions at 427.8 nm (Clilverd et al., 2017; Helliwell et al., 1973, 1980; Rogers & Honary, 2020; Rosenberg et al., 1977, 1971, 1980; Xu et al., 2020). Estimates of the D‐region changes are computed using the Sodankylä Ion and Neutral Chemistry (SIC) model designed for ionospheric D‐region studies (Turunen et al., 1996; Verronen et al., 2005). This 1‐D simulation code solved for the concentrations of 72 ions, including 29 negative ions, and 16 neutral species at altitudes across 20–150 km (Verronen et al., 2015). In the SIC model, about 400 chemical reactions are implemented, plus additional external forcing due to solar radiation (1–500 nm), electron and proton precipitation, and galactic cosmic radiation. Transport and chemistry are advanced in intervals of 5 s, matching the time step of the REDA precipitation fluxes. This model produces an ambient D‐region with additional ionization from the REDA induced electron precipitation. The neutral model atmosphere used for all simulations is MSISE‐00 (Picone et al., 2002).

**Figure 11:**
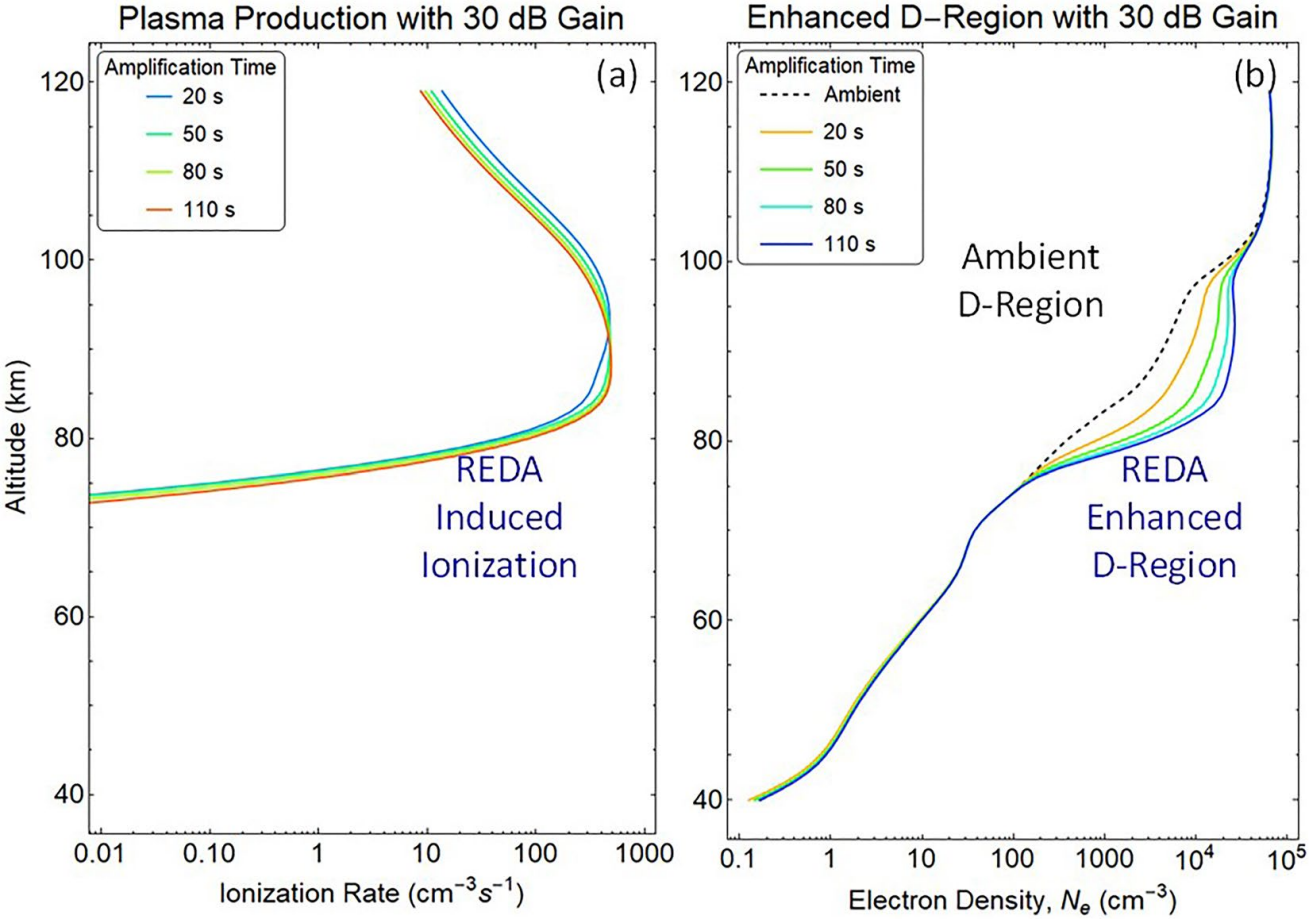
Predicted D‐region ionization caused by a 2 min Cygnus burn that strengthens very low frequency (VLF) whistlers by 30 dB through rocket exhaust driven amplification (REDA). The (a) artificial ionization is constrained between 73 and 120 km altitudes yielding (b) artificially enhanced D‐region densities in the 76–100 km range.

The precipitation flux associated with 30 dB REDA is predicted to produce enhanced electron and ion production in the D‐region through a process of inelastic electron‐neutral collisions (Figure 11a). The electron densities in the D‐region grow by a factor of 10 during the 2 min of the simulation (Figure 11b). The computed patch of ionization could produce measurable effects in both HF absorption of galactic radiation near 30 MHz (Rosenberg et al., 1977, 1980) and subionospheric VLF radio propagation in the EIWG (Helliwell et al., 1973).

**Figure 12:**
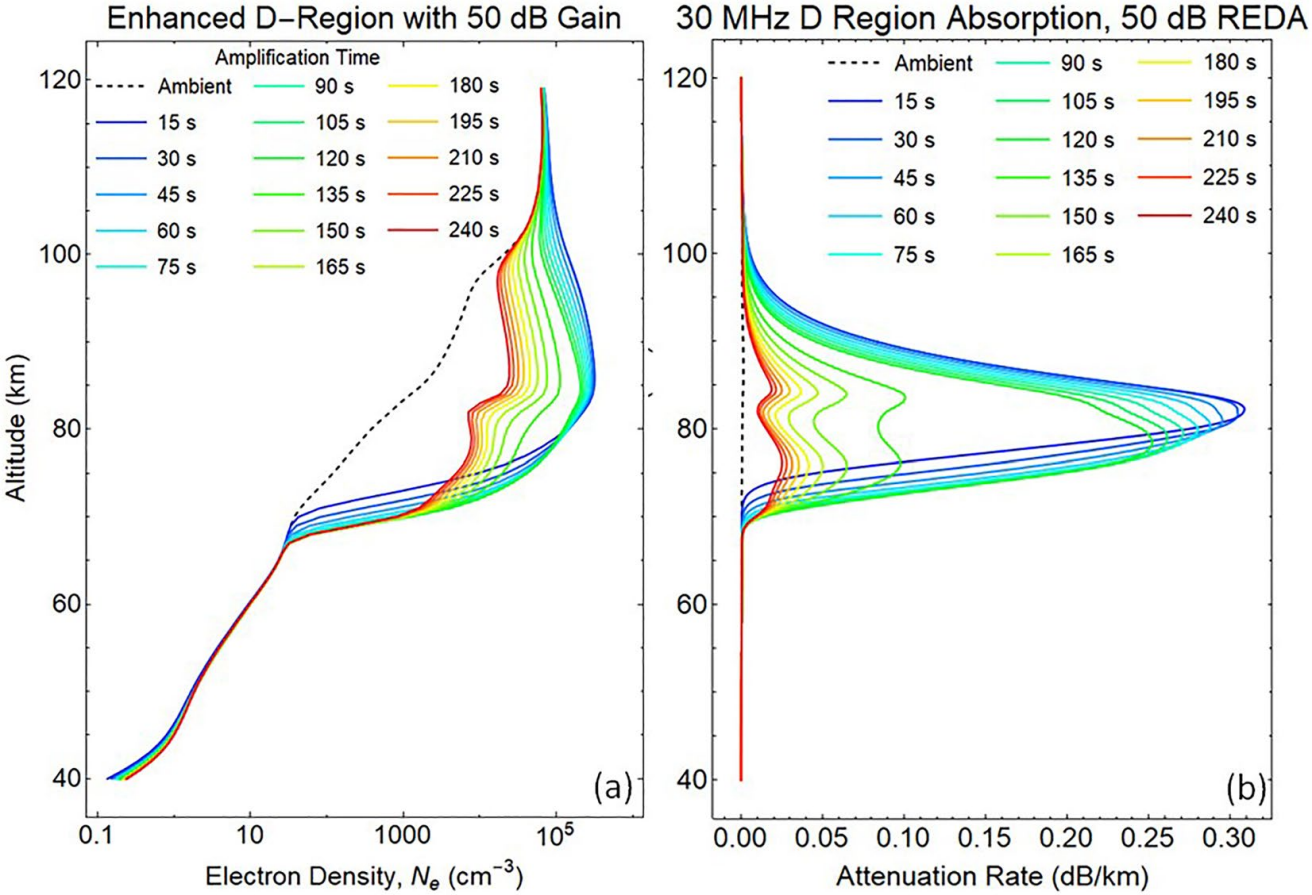
Ionization of the D‐region during a 50 dB rocket exhaust driven amplification (REDA) event. The precipitation flux shown in Figure 9 causes (a) D‐region growth by almost three orders of magnitude for 2 min and then relaxed toward the initial state after the REDA is over. The REDA process produces (b) an artificial layer extending from 68 to 110 km altitude that is responsible for strong attenuation of a 30 MHz signal propagating downward through the disturbed region.

If the whistler wave amplification reaches 50 dB, the REDA burn will have a much larger impact on the production of electrons and ions in the D‐region. Figure 12 shows the 120 s buildup during REDA followed by 120 s decay after the whistler amplification has stopped. The REDA effects on HF radio absorption are computed using wave attenuation rates for collisional plasmas (Rogers & Honary, 2020). D‐region enhancements are shown to linger in both the electron densities (Figure 12a) and the 30 MHz absorption profile (Figure 12b) as displayed over the 240 s period. It should be possible to detect the residual 30 MHz attenuation long after the enhanced electron precipitation fluxes have disappeared.

The D‐region ionization patch is 30 km thick, and it can extend in length horizontally for over 1,000 km along rocket engine burn trajectory. The width of the patch is typically 250 km depending on the spread of the rocket exhaust cloud (Bernhardt et al., 2021). The shape of the precipitation region is observable using ground diagnostics. An imaging riometer (Fiori & Danskin, 2016; Kavanagh et al., 2009; Moffat‐Griffin et al., 2010) could provide the dimensions of the absorption patch at the foot of the magnetic field lines. An incoherent scatter radar could also be used to estimate the size of the D‐Region patch (Kaeppler et al., 2020). Field line mapping could then determine the size of radiation belt regions impacted by the REDA process.

**Figure 13:**
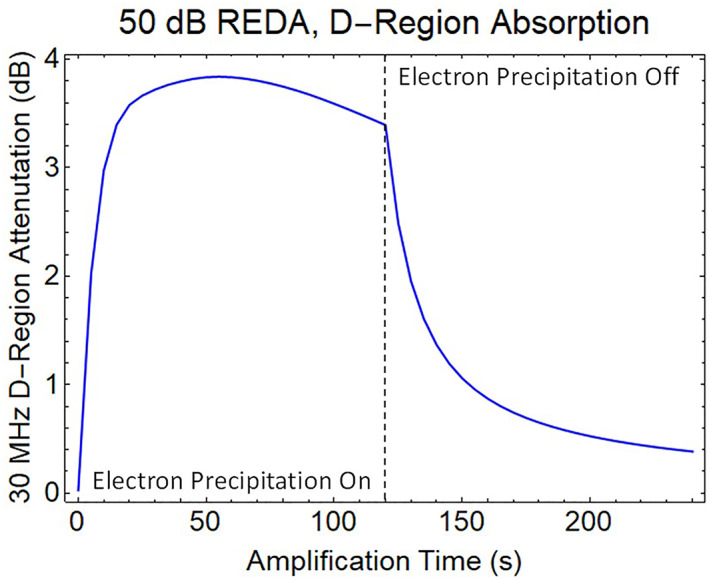
Computed riometer response to a finite duration period of rocket exhaust driven amplification (REDA) over a ground very low frequency (VLF) transmitter.

Height integration over a vertical absorption path though the attenuation profiles in Figure 12b provide a simulated time history of the radio amplitude signal available to a 30 MHz riometer (Figure 13). The precipitation event causes a rapid rise of radio absorption with a time constant of 6 s. During the first 120 s, production by collisional ionization and losses by electron‐ion recombination and electron attachment eventually approach equilibrium with a nearly constant absorption. The plasma decay time is about 18 s after the REDA event has finished.

**Figure 14:**
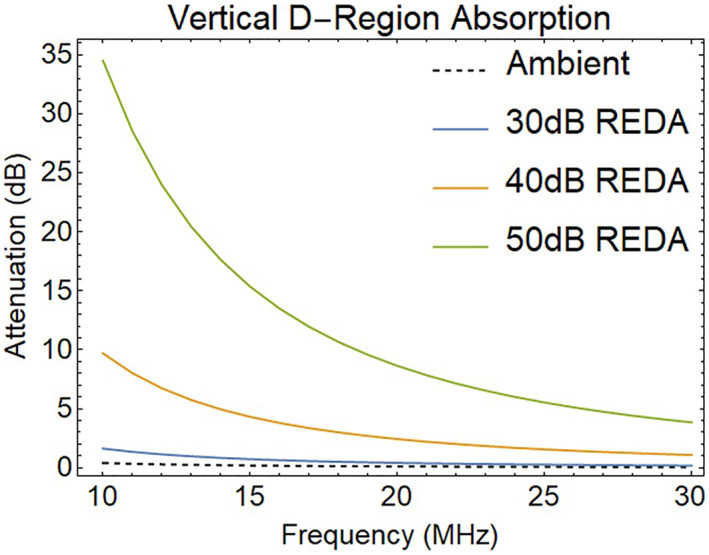
Computed impact of rocket exhaust driven amplification (REDA) gain on the vertical attenuation of HF signals during times of electron precipitation by amplified whistlers.

Using multiple frequencies, the electron‐density height profile may be determined by considering the rate of free‐electron production (artificial ionization rate) and the effective recombination rate (Kavanagh et al., 2009). Measurements at several frequencies can also provide estimates of the whistler mode amplification by the REDA process. The computed frequency dependence of HF equilibrium absorption for several levels of whistler wave amplification is given in Figure 14.

**Figure 15:**
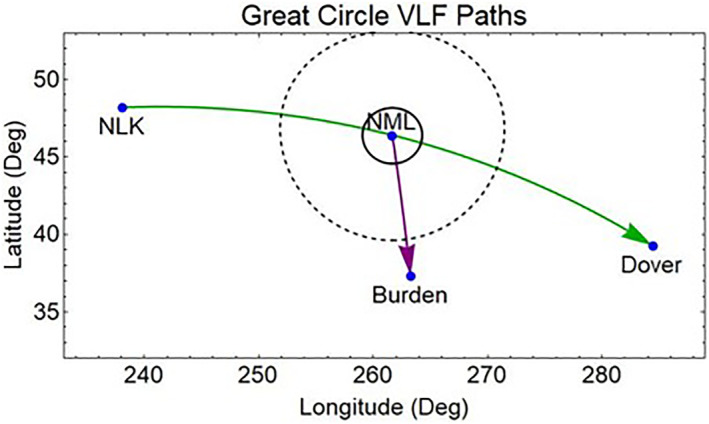
Very low frequency (VLF) propagation in the Earth‐ionosphere waveguide disturbed by rocket exhaust driven amplification (REDA) amplification and electron precipitation over the NML transmitter. The propagation model is used to estimate the VLF signal changes at Dover Delaware along the path from NLK that passes over NML. The NML signals are also simulated for reception at the Dover DE and Burden KS receives. The D‐region disturbance is assumed to have a fixed profile inside the solid circle centered at the NML transmitter.

Another method of detecting the effects of REDA is to measure the amplitude and phase changes in the EIWG for VLF signals propagating over long distances. Figure 15 illustrates the disturbed EIWG paths from a 24.8 kHz NLK transmitter near Seattle, Washington, and a 25.2 kHz NML transmitter at LaMoure, North Dakota to receivers in Dover DE and Burden KS. These sites are currently equipped for the VLF measurements of phase and amplitude disturbances associated with REDA (Gross et al., 2018).

**Figure 16:**
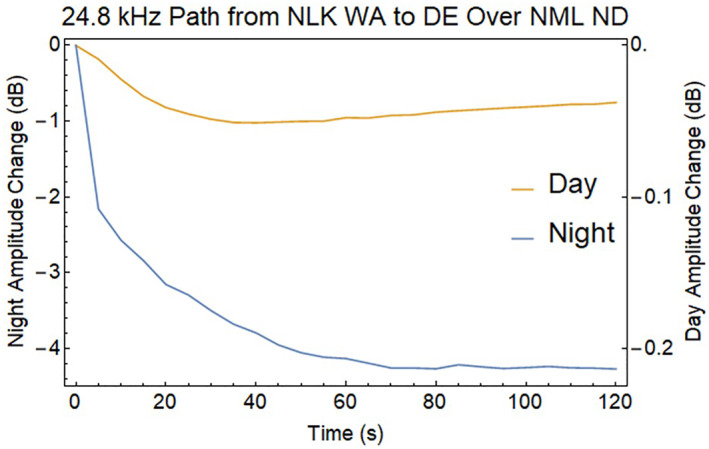
Drop in signal amplitude associated with very low frequency (VLF) propagation in the Earth‐ionosphere waveguide with a 50 dB rocket exhaust driven amplification (REDA) precipitation event at the center of the path. Note change in scale for the weak daytime disturbance.

**Figure 17:**
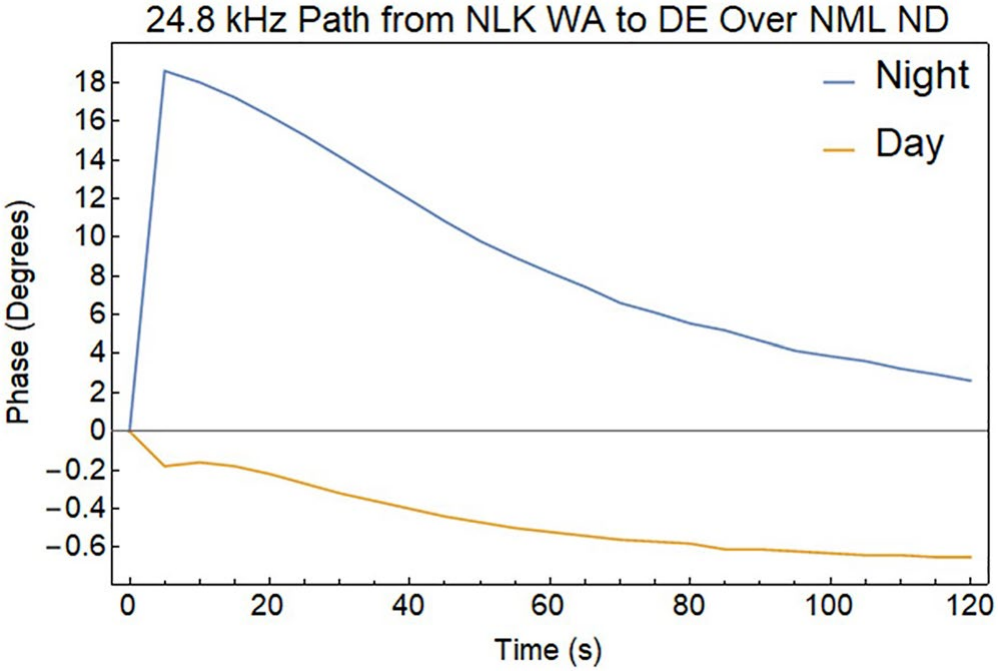
Shift in very low frequency (VLF) signal phase associated with propagation in the Earth‐ionosphere waveguide with a 50 dB rocket exhaust driven amplification (REDA) precipitation event at the center of the path. Note change in scale for the weak daytime disturbance.

The D‐region disturbances in middle of the EIWG path show changes in both phase and amplitude of the VLF magnetic field strength when encountering localized disturbances in the D‐layer. Predictions of these changes for EIWG propagation to a ground receiver were made using the Long Wavelength Propagation Capability model (Golkowski et al., 2021). The REDA impact calculation uses a great circle path between the 24.8 kHz NLK transmitter and a receiver in Delaware, which passes over the 25.2 kHz NML VLF transmitter. The radius of the enhanced D‐region is assumed to be 200 km around the location of REDA induced precipitation over the NML site. Both amplitude changes (Figure 16) and phase changes (Figure 17) are detectable, but the magnitudes of the REDA induced changes in the EIWG are much larger during the night.

**Figure 18:**
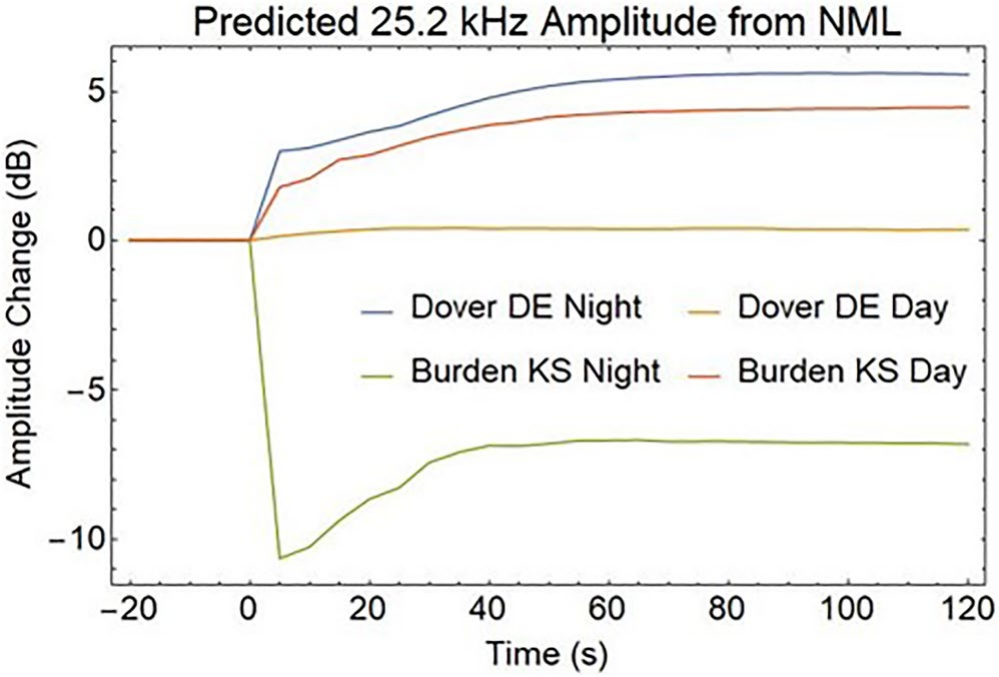
Amplitude changes for very low frequency (VLF) signals propagating from the source directly below the rocket exhaust driven amplification (REDA) burn to the two receiver locations in Figure 15. Because D‐region absorption is only found during the day, changes are stronger at night.

**Figure 19:**
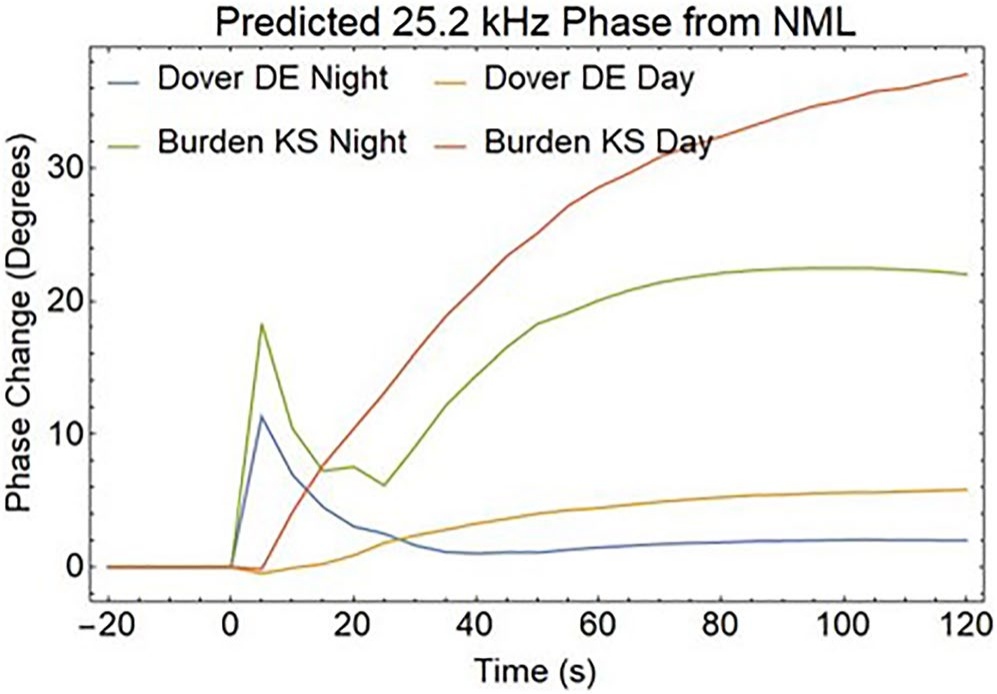
Phase changes for very low frequency (VLF) signals propagating from the source directly below the rocket exhaust driven amplification (REDA) burn to the two receiver locations in Figure 15. Nighttime phase changes show a transient pulse at the onset of REDA precipitation.

The VLF transmitter that excites the REDA will also excite the EIWG. The electron precipitation over the transmitter will impact the coupling to the waveguide. The amplitudes and phases computed for a 50 dB REDA event are shown in Figures 18 and 19, respectively. Rapid changes in both amplitude and phase are predicted for the strong REDA precipitation events. Multiple receiver measurements may be useful for determining the size of the modified D‐region.

**Figure 20:**
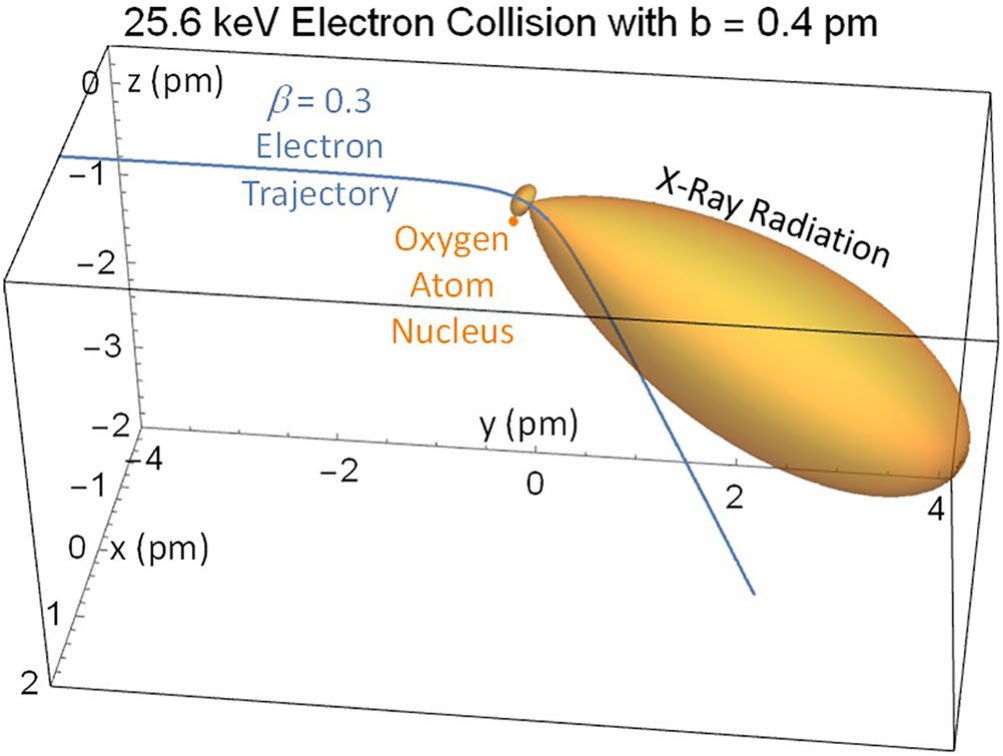
Bremsstrahlung X‐rays produced with a y‐directed energetic electron precipitated from the radiation belts by an amplified whistler signal.

The precipitating electrons can also excite bremsstrahlung X‐rays for observations by balloons. The X‐ray fluxes provide a diagnostic for the precipitating electron distributions (Xu & Marshall, 2019). The VLF waveguide and X‐ray experiments can be combined for detection of natural and artificial precipitation events (Rosenberg et al., 1971). The bremsstrahlung process of X‐ray emission generation is illustrated in Figure 20 by coulomb scatter from the atomic oxygen nucleus. The relativistic electrons travel along the magnetic field lines to enter the mesosphere where they encounter atmospheric constituents of oxygen and nitrogen. Electrons that pass near a positively charged nucleus emit bremsstrahlung radiation (Marshall et al., 2019; Rosenberg et al., 1971, 1980).

Predictions of the X‐ray fluxes from the 2 min REDA burn have been made using the GEANT4 model (Geant4 collaboration, 2012a, 2012b, 2013a, 2013b) to compute the time evolution of the X‐ray emission spectrum. The 50 dB REDA electron spectra shown in Figure 10 were approximated by piecewise constant energy spectra, with electrons at eight energy bands from 50 to 250 keV. For each of the eight electron energy bands, mono‐energetic electrons are emitted isotropically from 100 km altitude. They excite a spectrum of X‐rays over a range of energies at lower altitudes. The X‐ray photons at a fixed lower altitude were summed into 25 energy bins covering 10–250 keV to yield eight X‐ray flux vectors. The X‐ray spectrum from REDA electrons at a time step is a linear combination of the eight X‐ray flux vectors, for which the weights are a product of electron flux at the time step and an associated electron energy width.

**Figure 21:**
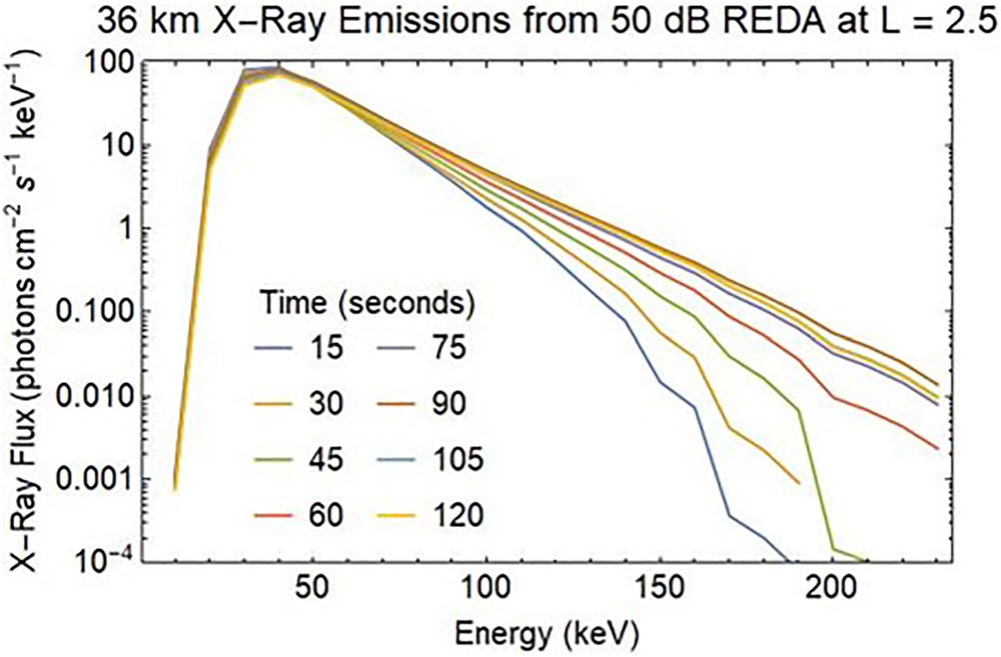
Bremsstrahlung X‐ray spectrum produced by precipitating electrons from a 50 dB rocket exhaust amplification of ambient whistler signals.

The computed X‐ray spectra are illustrated in Figure 21 for an altitude of 36 km. Initially, the X‐ray spectrum peaks at energies near 30–40 keV. Later for *t* > 90 s, the tail of the distribution grows, and the spectrum exponentially drops in energy as *I*_0_ exp(−*E*/*E*_1_) with *E*_1_ = 17 keV. The curves in Figure 19 can be used to estimate X‐ray intensities for balloon‐based sensors designed to show the effectiveness of REDA of whistlers for scattering radiation belt particles.

The computed X‐ray spectra are illustrated in for an altitude of 36 km. Initially, the X‐ray spectrum peaks at energies near 30–40 keV. Later for t > 90 s, the tail of the distribution grows, and the spectrum exponentially drops in energy as I_0_ exp(−*E*/*E*_1_) with *E*_1_ = 17 keV. The curves in can be used to estimate X‐ray intensities for balloon‐based sensors designed to show the effectiveness of Rocket Exhaust Driven Amplification of whistlers for scattering radiation belt particles.

**Figure 22:**
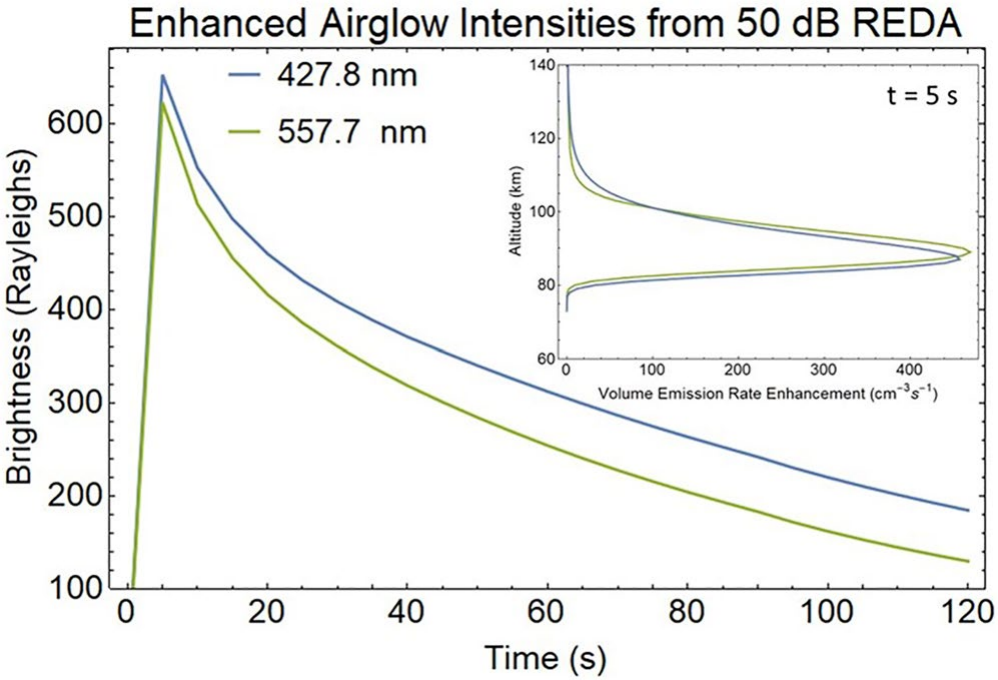
Artificial aurora intensities from rocket exhaust driven amplification (REDA) induced electron precipitation obtained by vertical integration of the enhanced volume emission rate profile (inset) at each time.

It has been long known that VLF wave events can be associated with transient optical emissions (Helliwell & Mende, 1980). Energetic electron precipitation fluxes from the 50 dB REDA process are input to the Global Airglow (GLOW; Soloman, 2017) to calculate mesospheric and thermospheric airglow emissions. Volume emission rates are obtained by integrating through the model output fields yielding the vertical column brightness. Emissions subject to absorption or scattering use radiative transfer calculations. The standard GLOW driver code was modified to use altitude ranges above 73 km driven by the incoming fluxes given in Figure 10. The computed total height integrated intensities, in Rayleighs, of the emission profiles of 557.7 nm (green) and 427.8 nm (blue) are shown in Figure 22. The N_2_(^1^P) 673.0 nm emission should also be produced. Profiles of the volume emission rates show that the emissions from O(^1^S) and N_2_^+^ initially range from 85 to 105 km altitude and drop by 10 km at the end of the simulation. Imaging of these optical emissions are a primary diagnostic to determine the spatial extent of the REDA wave interactions in the radiation belts.

## 5 Conclusions and Future Research

A new technique has been developed for intensification of low‐frequency electromagnetic waves in space plasmas. The amplification process involves propagation through a medium that has been activated by the injection of hypersonic water vapor from a rocket nozzle. Charge exchange between the water molecules and thermal oxygen ions yields a beam of energetic water ions. If the injection is perpendicular to the ambient magnetic field, a ring‐beam distribution is produced in the ions that excites high amplitude lower‐hybrid waves over a large frequency spectrum. The lower hybrid oscillations serve as a pump for a parametric amplifier of existing whistler or EMIC waves. The WTWPA transfers energy from the lower hybrid pump wave to the whistler signal and a lower hybrid idler wave by resonance matching (Bernhardt, 2021).

REDA of coherent whistler waves in the topside ionosphere is an example of the WTWPA that has been experimentally verified (Bernhardt et al., 2021). At the conclusion of the NG‐13 flight of the Cygnus satellite to the ISS, the 25.2 kHz transmissions from the Navy VLF site NML were amplified by 30 dB using a 60 s burn of the BT‐4 main engine. Measurements taken 500 km from the burn by the RRI wave sensor on the SWARM‐E spacecraft demonstrated that the 8 pT wave was amplified to 270 pT, making it the strongest man‐made, coherent VLF signal ever observed in space. This indicates that the key to creation of intense whistler waves may *not* be by generating them in space, but with in situ *amplification* of ground generated VLF waves.

The strongest VLF waves from ground‐based transmitters are observed in space at night because of daytime D‐Region absorption (Němec et al., 2020). Whistler modes from near‐Earth sources are much weaker during daylight periods and do not have as strong interaction with the radiation belts. The REDA technique may intensify the daytime VLF waves in space to intensities larger than normally found at night so future REDA experiments should be conducted at all local times.

**Figure 23:**
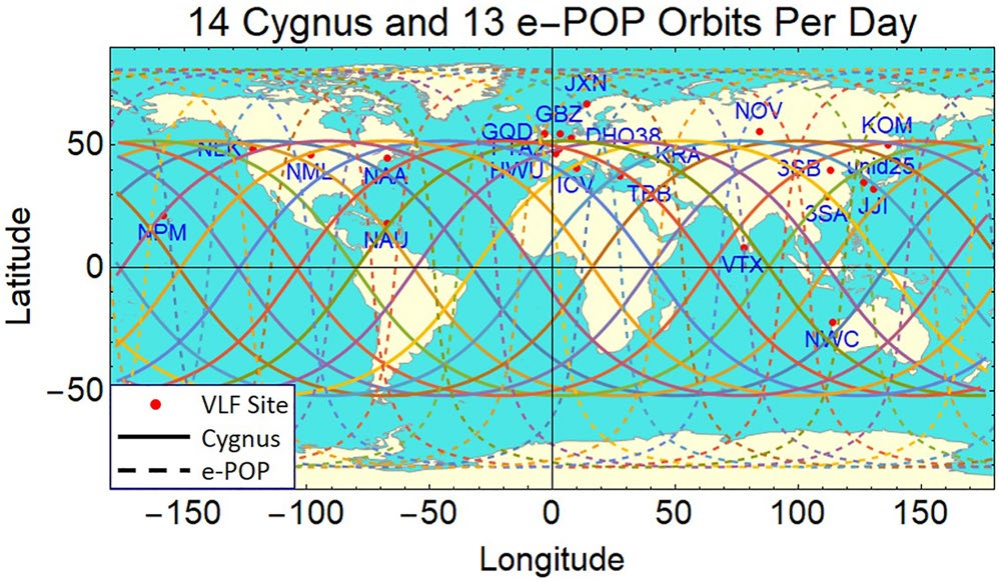
Cygnus and SWARM‐E/e‐POP orbits for rocket exhaust driven amplification (REDA) burn experiments over existing very low frequency (VLF) communications transmitters around the world.

Experiments are currently being planned to verify and optimize the REDA concept with dedicated Cygnus burns and other spacecraft in orbit over ground‐based VLF transmitters. Figure 23 shows a map of the 21 existing VLF transmitters around the world used for communications applications. These transmitters excite the Earth ionosphere waveguide, which causes leakage of signals into the ionosphere as whistler modes (Y. Kasahara et al., 2018). A superimposed orbit of the Cygnus satellite (solid line) with an inclination that is optimum for rocket burns directly over the ground‐based VLF sites is also shown. The requirement of exhaust injection perpendicular to the ambient magnetic field is easily satisfied because most orbits are traveling from west to east perpendicular to the ambient north‐south orientation of the magnetic meridian. Additional flexibility in the experiments is available since the burns may occur over the geomagnetic conjugate location to the ground‐based transmitter. Diagnostics of REDA experiments can be supported by existing networks of ground‐based instruments such as VLF receivers, auroral imagers, and sampling riometers as described by Shiokawa et al. (2017).

**Figure 24:**
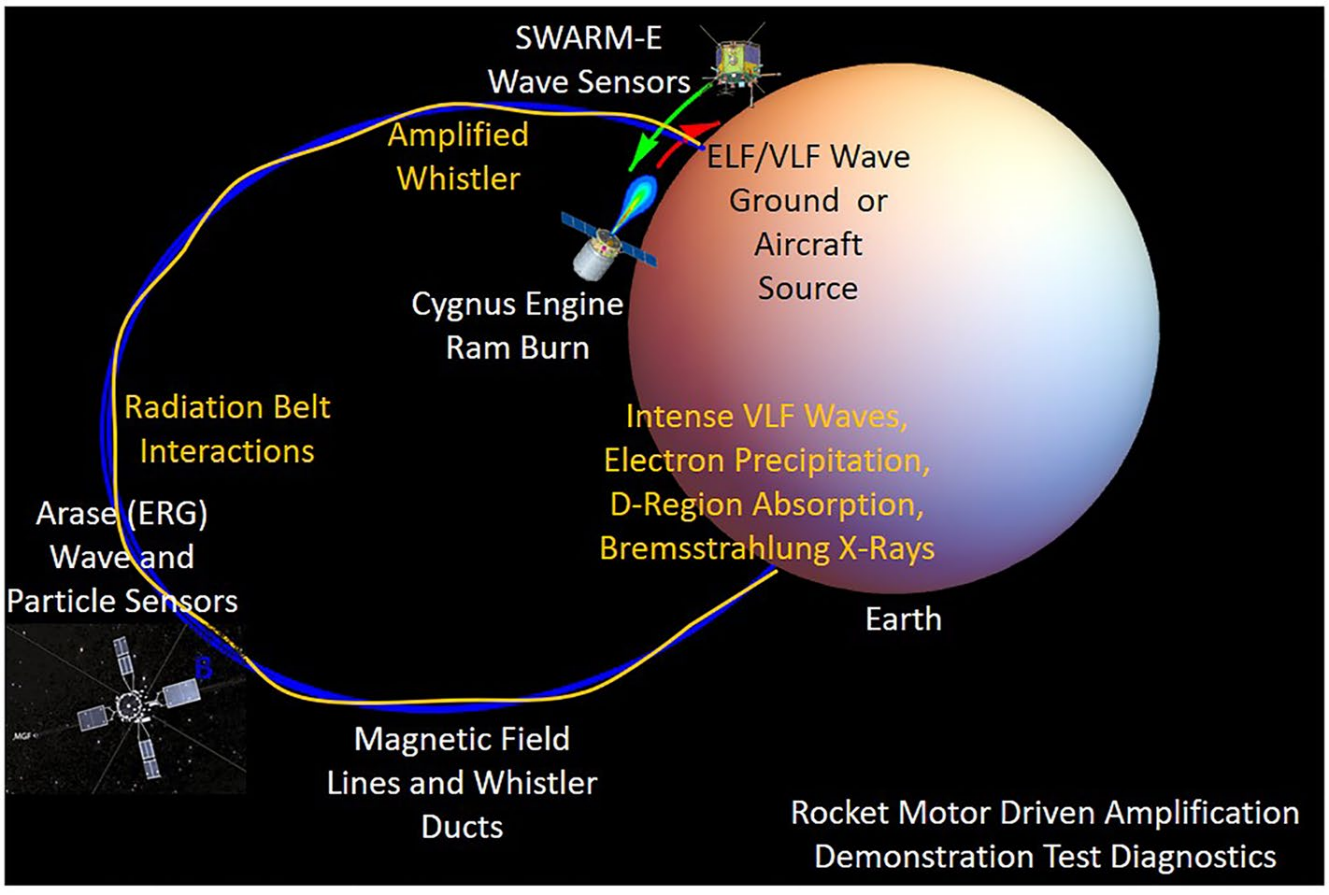
Proposed configuration for rocket exhaust driven amplification (REDA) demonstration and concept validation. The test diagnostics will measure amplified whistlers, scatter of energetic particles, enhanced plasma from electron precipitation, mesospheric airglow, and X‐ray emissions in the stratosphere.

Along with SWARM‐E in low‐earth‐orbit, satellites such as ARASE (ERG) in mid‐earth‐orbit can support the REDA measurements with a complete suite of wave and particle instruments (S. Kasahara et al., 2018; Y. Kasahara et al., 2018; Kazama et al., 2017). A diagram of an experimental geometry for future REDA tests is given in Figure 24. A field line associated with a ground‐based VLF transmitter would be the center for a region of ambient whistler signals located 500 km in radius near the Earth. The experiment would setup as many space‐based and ground‐based instruments along this field line during a dedicated Cygnus burn. The results of these experiments will depend on several factors including (a) the presence of field‐aligned enhancement ducts to guide the intense whistler into the equatorial regions of the magnetosphere, (b) orientation of the rocket engine relative to the spacecraft orbit vector, (c) burn duration and flow rate of the rocket motor, and (d) broadcast power of the ground‐based source. Unlike natural events, the REDA experiments have predictable locations and times of the precipitation events. Large VLF communications systems (Kulkarni et al., 2008) and high‐power HF modulations of the high latitude electrojet (Lehtinen & Inan, 2008) can be used as sources for the VLF waves. These experiments will be supported by theoretical efforts using both fluid and kinetic theory for the theory and diagnostic simulation models presented here.

## Data Availability

All data used in this article are available from the e‐POP Data Center at https://epop-data.phys.ucalgary.ca/. The RBSPICE data were obtained from http://rbspicea.ftecs.com.
